# Supplementary material for: Dynamics and control of typhoid fever in Sheno town, Ethiopia: A comprehensive nonlinear model for transmission analysis and effective intervention strategies
Source: PLoS One. 2024 Aug 7;19(8):e0306544. doi: 10.1371/journal.pone.0306544 (PMC11305579; doi:10.1371/journal.pone.0306544)
Supplement: Attachment — Submitted filename: makinde.pdf [file pone.0306544.s001.pdf]

makinde

by John Akanni

Submission date: 26-Dec-2023 02:04PM (UTC+0100)
Submission ID: 1843223552
File name: PONE-D-23-42909_reviewer.pdf (987.25K)
Word count: 7527
Character count: 36993

PLOS ONE

Dynamics and Control of Typhoid Fever in Sheno Town: A Comprehensive Nonlinear Model for Transmission Analysis and Effective Intervention Strategies

--Manuscript Draft--

**Manuscript Number:**	PONE-D-23-42909
**Article Type:**	Research Article
**Full Title:**	Dynamics and Control of Typhoid Fever in Sheno Town: A Comprehensive Nonlinear Model for Transmission Analysis and Effective Intervention Strategies
**Short Title:**	Dynamics and Control of Typhoid Fever in Sheno Town
**Corresponding Author:**	Belela Samuel Kotola, Ph.D<br>Oda Bultum University<br>Addis Ababa, Oromia ETHIOPIA
**Keywords:**	Typhoid Fever; Mathematical Model; Sheno Town and Intervention Strategies
**Abstract:**	In this study, we propose a robust nonlinear SICRS (Susceptible-Infected-Carrier-Recovered) mathematical model to comprehensively analyze the dynamics of typhoid fever within the Sheno town community. Our investigation includes a thorough qualitative analysis, encompassing solution positivity, solution region boundedness, and stability assessments for both disease-free and endemic equilibrium points, utilizing the Routh-Hurwitz stability criterion. Employing the next-generation matrix, we determine the basic reproduction number of the system, revealing its numerical value to be greater than one a clear indication of typhoid fever transmission in Sheno town. Our findings demonstrate the instability of the disease-free equilibrium point and the stability of the endemic equilibrium point. To propose effective control measures, we identify two critical parameters the infectious rate and the recruitment rate . To maintain a basic reproduction number below one and effectively control the disease, it is crucial to uphold parametric values and . The impact of other parameters on the basic reproduction number is thoroughly discussed in their respective subsections. This research provides valuable insights into the dynamics of typhoid fever and suggests targeted strategies for its control within the Sheno town community.
**Order of Authors:**	Belela Samuel Kotola, Ph.D
	Lema Abdela Baisa, Ph.D. candidate
**Additional Information:**	
**Question**	**Response**
**Financial Disclosure**<br><br>Enter a financial disclosure statement that describes the sources of funding for the work included in this submission. Review the submission guidelines for detailed requirements. View published research articles from *PLOS ONE* for specific examples.<br><br>This statement is required for submission and **will appear in the published article** if the submission is accepted. Please make sure it is accurate.	The author(s) received no specific funding for this work.

Powered by Editorial Manager® and ProduXion Manager® from Aries Systems Corporation

**Funded studies**<br>Enter a statement with the following details:<br>• Initials of the authors who received each award<br>• Grant numbers awarded to each author<br>• The full name of each funder<br>• URL of each funder website<br>• Did the sponsors or funders play any role in the study design, data collection and analysis, decision to publish, or preparation of the manuscript?<br><br>Did you receive funding for this work?	
**Competing Interests**<br><br>Use the instructions below to enter a competing interest statement for this submission. On behalf of all authors, disclose any competing interests that could be perceived to bias this work—acknowledging all financial support and any other relevant financial or non-financial competing interests.<br><br>This statement is required for submission and **will appear in the published article** if the submission is accepted. Please make sure it is accurate and that any funding sources listed in your Funding Information later in the submission form are also declared in your Financial Disclosure statement.<br><br>View published research articles from *PLOS ONE* for specific examples.	The authors have declared that no competing interests exist

Powered by Editorial Manager® and ProduXion Manager® from Aries Systems Corporation

**NO authors have competing interests**<br><br>Enter: *The authors have declared that no competing interests exist.*<br><br>**Authors with competing interests**<br><br>Enter competing interest details beginning with this statement:<br><br>*I have read the journal's policy and the authors of this manuscript have the following competing interests: [insert competing interests here]*<br><br>* typeset	
**Ethics Statement**<br><br>Enter an ethics statement for this submission. This statement is required if the study involved:<br><br>• Human participants<br>• Human specimens or tissue<br>• Vertebrate animals or cephalopods<br>• Vertebrate embryos or tissues<br>• Field research<br><br>Write "N/A" if the submission does not require an ethics statement.<br><br>General guidance is provided below. Consult the submission guidelines for detailed instructions. **Make sure that all information entered here is included in the Methods section of the manuscript.**	N/A

Powered by Editorial Manager® and ProduXion Manager® from Aries Systems Corporation

**Format for specific study types**<br><br>**Human Subject Research (involving human participants and/or tissue)**<br>• Give the name of the institutional review board or ethics committee that approved the study<br>• Include the approval number and/or a statement indicating approval of this research<br>• Indicate the form of consent obtained (written/oral) or the reason that consent was not obtained (e.g. the data were analyzed anonymously)<br><br>**Animal Research (involving vertebrate animals, embryos or tissues)**<br>• Provide the name of the Institutional Animal Care and Use Committee (IACUC) or other relevant ethics board that reviewed the study protocol, and indicate whether they approved this research or granted a formal waiver of ethical approval<br>• Include an approval number if one was obtained<br>• If the study involved *non-human primates*, add *additional details* about animal welfare and steps taken to ameliorate suffering<br>• If anesthesia, euthanasia, or any kind of animal sacrifice is part of the study, include briefly which substances and/or methods were applied<br><br>**Field Research**<br><br>Include the following details if this study involves the collection of plant, animal, or other materials from a natural setting:<br>• Field permit number<br>• Name of the institution or relevant body that granted permission	
**Data Availability**<br><br>Authors are required to make all data underlying the findings described fully available, without restriction, and from the time of publication. PLOS allows rare exceptions to address legal and ethical concerns. See the PLOS Data Policy and FAQ for detailed information.	Yes - all data are fully available without restriction

Powered by Editorial Manager® and ProduXion Manager® from Aries Systems Corporation

A Data Availability Statement describing where the data can be found is required at submission. Your answers to this question constitute the Data Availability Statement and **will be published in the article**, if accepted.<br><br>**Important:** Stating 'data available on request from the author' is not sufficient. If your data are only available upon request, select 'No' for the first question and explain your exceptional situation in the text box.<br><br>Do the authors confirm that all data underlying the findings described in their manuscript are fully available without restriction?	
**Describe where the data may be found in full sentences. If you are copying our sample text, replace any instances of XXX with the appropriate details.**<br><br>• If the data are **held or will be held in a public repository**, include URLs, accession numbers or DOIs. If this information will only be available after acceptance, indicate this by ticking the box below. For example: *All XXX files are available from the XXX database (accession number(s) XXX, XXX.*).<br>• If the data are all contained **within the manuscript and/or Supporting Information files**, enter the following: *All relevant data are within the manuscript and its Supporting Information files.*<br>• If neither of these applies but you are able to provide **details of access elsewhere**, with or without limitations, please do so. For example:<br><br>*Data cannot be shared publicly because of [XXX]. Data are available from the XXX Institutional Data Access / Ethics Committee (contact via XXX) for researchers who meet the criteria for access to confidential data.*<br><br>*The data underlying the results presented in the study are available from (include the name of the third party*	no data available

Powered by Editorial Manager® and ProduXion Manager® from Aries Systems Corporation

and contact information or URL).<br>• This text is appropriate if the data are owned by a third party and authors do not have permission to share the data.<br><br>* typeset	
Additional data availability information:	

Powered by Editorial Manager® and ProduXion Manager® from Aries Systems Corporation

Click here to access/download;Manuscript;Lemma Article1.docx

Dynamics and Control of Typhoid Fever in Sheno Town: A Comprehensive Nonlinear Model for Transmission Analysis and Effective Intervention Strategies

Lema Abdela Baisa[1,2] Belela Samuel Kotola[2]

[1]Department of Mathematics, Oda Bultum University, Chiro, Ethiopia
[2]Department of Mathematics, Debre Birhan University, Debre Birhan, Ethiopia

ABSTRACT

In this study, we propose a robust nonlinear SICRS (Susceptible-Infected-Carrier-Recovered) mathematical model to comprehensively analyze the dynamics of typhoid fever within the Sheno town community. Our investigation includes a thorough qualitative analysis, encompassing solution positivity, solution region boundedness, and stability assessments for both disease-free and endemic equilibrium points, utilizing the Routh-Hurwitz stability criterion. Employing the next-generation matrix, we determine the basic reproduction number of the system, revealing its numerical value to be greater than one a clear indication of typhoid fever transmission in Sheno town. Our findings demonstrate the instability of the disease-free equilibrium point and the stability of the endemic equilibrium point. To propose effective control measures, we identify two critical parameters the infectious rate (α) *and the recruitment rate* (n)*. To maintain a basic reproduction number below one and effectively control the disease, it is crucial to uphold parametric values* $\alpha < 0.0016$ *and* $n < 0.0179$*. The impact of other parameters on the basic reproduction number is thoroughly discussed in their respective subsections. This research provides valuable insights into the dynamics of typhoid fever and suggests targeted strategies for its control within the Sheno town community.*

Key words*: Typhoid Fever, Mathematical Model, Sheno Town and Intervention Strategies.*

1. Introduction

Throughout history, human populations have experienced major epidemics of infectious diseases, often resulting in large numbers of deaths, panic, disruption of trade, and political instability [1]. Typhoid Fever is one of the most dangerous human infectious diseases caused by a bacterium called salmonella typi (S.typi). It belongs to the family Enterbacteriaceae; members of this family are salmonella paratyphoid A, salmonella paratyphi B, and salmonella choleraesurs and salmonella type [2]. The bacterium then multiplies in the cells of the organs and re-enters the blood bladder, biliary system and the lymphatic tissue of the bowel, then multiply in high numbers. The incubation period for typhoid fever is eight to fourteen days and the duration of illness is about four to six weeks. The patient or infected humans show the following sign and symptoms; sustained fever, poor appetite, vomiting, severe headache, constipation, malaise, fatigue and non-productive cough can occur in the early stage. Intensity of fever and abdominal pain increases and diarrhea follows[2]. Humans are the only natural host and reservoir. The infection is transmitted by ingestion of food or water contaminated with faeces. Ice cream is recognized as a significant risk factor for the transmission of typhoid fever. Shellfish taken from contaminated water, and raw fruit and vegetables fertilized with sewage, have been sources of past outbreaks.

Mathematical modeling of infectious diseases has become a key tool in order to understand, predict and control the spread of infections. The fundamental difference to chronic disease epidemiology is that the temporal aspect is paramount. The aim of epidemic modeling is thus to model the spread of a disease in a population made up of a (possibly large) integer number of individuals[3]. To simplify the description of this population, it is common to use a compartmental approach to modeling for instance in its simplest form the population is divided into classes of susceptible, infective and recovered individuals. Disease dynamics can then be characterized by a mathematical description of each individual's transitions between compartments, subject to the state of the remaining individuals [4, 5].

In Ethiopia, as in other developing countries, it is difficult to evaluate the burden of salmonellosis because of the limited scope of studies and lack of coordinated epidemiological surveillance systems. In addition, under-reporting of cases and the presence of other diseases considered to be of high priority may have overshadowed the problem of salmonellosis. The real situation of

antibiotic resistance is also not clear since Salmonella are not routinely cultured and their resistance to antibiotics cannot be tested. As in a developed country, however, to control the spread of salmonellosis, surveillance for Salmonella serovars and the assessment of antimicrobial susceptibility is essential. In Ethiopia there have been several studies conducted on salmonellosis which suggest an increase in the antibiotic resistance of Salmonella to commonly used antimicrobials in both the public health and veterinary sectors. With the exception of S.typi, bacterial identification is usually to sero group level, and sometimes only to genus level. Because of this, Paratyphoid B and C isolates are reported with other sero group B and C members and it is impossible to differentiate typhoidal from non-typhoidal salmonellae. So far, there is only a single published study, which uses serotyping to describe the different serovars of Salmonella circulating in Ethiopia [6].

2. Baseline Model Formulation

We study the spread and control of typhoid fever by extending the model in [1], by incorporating new assumptions considered as "A successfully treated individual can become infected again and new born individuals inter in to the susceptible group." That is individual who recovered from typhoid fever can be infected again by typhoid fever[7]. So the population from recovered group entering in to susceptible class by the rate k. and newborn individuals inter in to susceptible group by net birth rate n.

Basic Assumption: The following assumptions are included in the extended model.

- Susceptible ingest solid or liquid food, a proportion of which are infective. Assume that this proportion is equal to the prevalence of infective in the population.
- All susceptible individuals are equally susceptible, all infected individuals are equally infectious and all carriers are equally infectious too.
- The disease is transmitted by direct transmission or by ingestion of contaminated food or liquid by susceptible individuals.
- There is no latent period for the disease; hence the disease is transmitted instantaneous on contact.
- The natural death rate in all compartments is considered as equal.
- There is no immigration and migration in the population.

- New born individual inter in to susceptible group by net birth rate n.
- A successfully treated individual can become infected again.
- Assume that the typhoid induced death rate and carrier induced death rate are equal.

The flow diagram of the model is given in Fig.1 below.

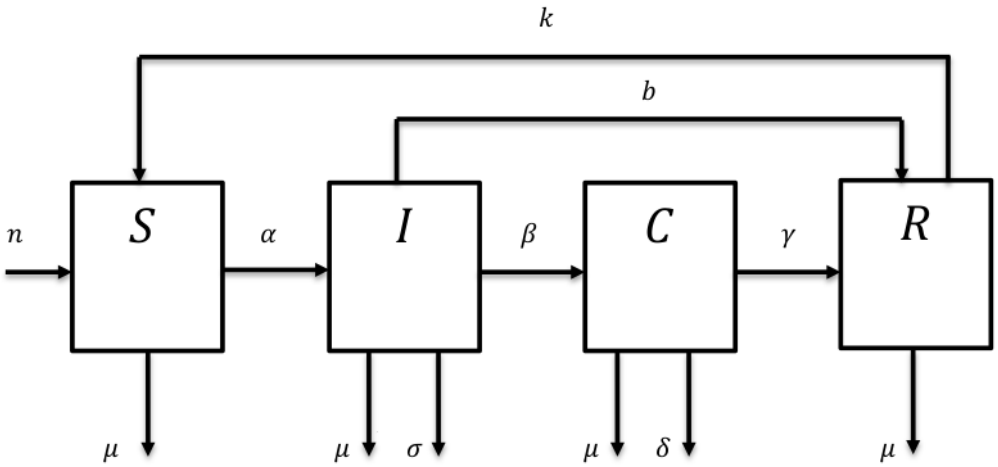

Fig.1 Flow diagram of SICRS model

The corresponding mathematical model equations are:

$$\frac{dS}{dt} = n - \alpha SI - \mu S + kR \ldots \ldots \ldots \ldots \ldots \ldots (5)$$

$$\frac{dI}{dt} = \alpha SI - (\mu + \sigma)I - \beta I - bI \ldots \ldots \ldots \ldots \ldots \ldots (6)$$

$$\frac{dC}{dt} = \beta I - (\mu + \delta)C - \gamma C \ldots \ldots \ldots \ldots \ldots \ldots (7)$$

$$\frac{dR}{dt} = bI + \gamma C - \mu R - kR \ldots \ldots \ldots \ldots \ldots \ldots (8)$$

with initial condition $S(0) = S_0 > 0, I(0) = I_0 \geq 0, C(0) = C_0 \geq 0$ and $R(0) = R_0 \geq 0$.

The state variables and the parameters of the model are described in Table 1below.

Variables and Parameters	Description
**S**	Susceptible
**I**	Infectious
**C**	Carrier
**R**	Recovered
n	Recruitment rate in the population
μ	Natural death rate
α	The rate of infectious
σ	The typhoid fever –indicated mortality rate
β	The rate of progression from infective to carrier
δ	The carrier-induced mortality rate
γ	The rate of recovery from the carrier stage
b	The rate of recovery from infectious stage
k	Re-susceptibility rate or the rate of recovered population being susceptible again

Table 1: The description of the variables and parameters of the model.

2.1 Positivity of the Solutions

For a dynamical system of model to be epidemiologically meaningful, it is important to show that all the solutions with non-negative initial conditions will remain non-negative. To show this we have taken each differential equation of the dynamical system given above and showed that their solutions are non–negative as follows.

Let us take the first differential equation

$$\frac{dS}{dt} = n - \alpha SI - \mu S + kR$$

Then by using, the integrating factor $e^{\int_0^\tau (\alpha I+\mu)dt}$ we obtained the solution;

$$S(t) = e^{-\int_0^\tau (\alpha I+\mu)dt}\left[\int_0^\tau (n + kR)e^{\int_0^\tau (\alpha I+\mu)dt}dt + S(0)\right]$$

$$\Rightarrow S(t) = S_0 e^{-\int_0^\tau (\alpha I+\mu)dt} + \left[(n + kR)e^{-\int_0^\tau (\alpha I+\mu)dt}\right]\int_0^\tau e^{\int_0^\tau (\alpha I+\mu)dt}dt$$

In this solution, $n + kR$ is positive, s_0, the initial susceptible population is also positive and the range of exponential function is positive.

This implies that $S(t)$ is positive.

Following the same procedures, the solution of all state variables are positive.

2.2 Boundedness of the Solution

To show the boundednes of the solution, we have to show lower bound and upper bound.

As a result, $N(0) = N_0 > 0, S(0) = S_0 > 0, I(0) = I_0 \geq 0, C(0) = C_0$ and $R(0) = R_0 \geq 0$.

Those initial conditions considered being lower bound. Now we are going to show upper bound. By taking the relation $N(t) = S(t) + I(t) + C(t) + R(t)$ and differentiating both sides of the equation with respect to time, we *get* $\frac{dN}{dt} = \frac{dS}{dt} + \frac{dI}{dt} + \frac{dC}{dt} + \frac{dR}{dt}$

$$\Rightarrow \frac{dN}{dt} = (n - \alpha SI - \mu S + kR) + (\alpha SI - (\mu + \sigma)I - \beta I - bI) + (\beta I - (\mu + \delta)C - \gamma C) + (bI + \gamma C - \mu R - kR)$$

$$\Rightarrow \frac{dN}{dt} = n - N\mu - \sigma I - \delta c, \; since \; N = S + I + C + R$$

$$\Rightarrow \frac{dN}{dt} + N\mu = n - \sigma I - \delta c$$

Since, we are assumed that the typhoid- induced death rate and carrier-induced death rate are equal, That is $\delta = \sigma$

$$\Rightarrow \frac{dN}{dt} + N\mu = n - \sigma(I + C)$$

$$\Rightarrow \frac{dN}{dt} + (\mu + \sigma)N = n + \sigma(S + R)$$

This is the form of first order linear ordinary differential equation.

$$N(t) = N_0 e^{-\int_0^\tau (\mu+\sigma)dt} + \left[(n + \sigma(S + R)e^{-\int_0^\tau (\mu+\sigma)dt}\right] \int_0^\tau e^{\int_0^\tau (\mu+\sigma)dt} dt$$

Since, any exponential function with positive coefficients is always positive, $N(t)$ is positive. That is the solution, $N(t)$ is bounded above by

$$N_0 e^{-\int_0^\tau (\mu+\sigma)dt} + [(n + \sigma(S + R)]e^{-\int_0^\tau (\mu+\sigma)dt} \int_0^\tau e^{\int_0^\tau (\mu+\sigma)dt} dt.$$

Hence $N_0 \leq N \leq N_0 e^{-\int_0^\tau (\mu+\sigma)dt} + [(n + \sigma(S + R)]e^{-\int_0^\tau (\mu+\sigma)dt} \int_0^\tau e^{\int_0^\tau (\mu+\sigma)dt} dt$

3. Equilibrium Points

3. 1. The Disease Free Equilibrium Point (DFEP)

To determine disease free equilibrium point, equating equation $(5) - (8)$ equal to zero as follows:

Therefore the disease free equilibrium point $(S^0, I^0, C^0, R^0) = \left(\frac{n}{\mu}, 0,0,0\right)$.

3. 2. Endemic Equilibrium Point (EEP)

To determine endemic equilibrium point, equating the equation $(5)-(8)$ equal to zero, where $I \neq 0$. That is $\frac{dS}{dt}=0, \frac{dI}{dt}=0, \frac{dC}{dt}=0$ and $\frac{dR}{dt}=0$.

$$\Rightarrow S=\frac{\mu+\sigma+\beta+b}{\alpha} \ldots\ldots\ldots (9)$$

$$\Rightarrow I=\left(\frac{\mu+\delta+\gamma}{\beta}\right) C \ldots\ldots\ldots (10)$$

$$\Rightarrow R=\left(\frac{b(\mu+\delta+\gamma)+\beta\gamma}{\beta(\mu+k)}\right) C \ldots\ldots\ldots (11)$$

$$\Rightarrow C=\frac{\beta(\mu+k)[\mu(\mu+\sigma+\beta+b)-\alpha n]}{\alpha[bk(\mu+\delta+\gamma)+k\beta\gamma-(\mu+k)(\mu+\sigma+\beta+b)(+\delta+\gamma)]} \ldots\ldots\ldots (12)$$

$$\Rightarrow I=\frac{(\mu+k)(\mu+\delta+\gamma)[\mu(\mu+\sigma+\beta+b)-\alpha n]}{\alpha[(bk(\mu+\delta+\gamma)+k\beta\gamma)-(\mu+k)(\mu+\sigma+\beta+b)(+\delta+\gamma)]} \ldots\ldots\ldots (13)$$

$$\Rightarrow R=\frac{(b(\mu+\delta+\gamma)+\beta\gamma)[\mu(\mu+\sigma+\beta+b)-\alpha n]}{\alpha[(bk(\mu+\delta+\gamma)+k\beta\gamma)-(\mu+k)(\mu+\sigma+\beta+b)(+\delta+\gamma)]} \ldots\ldots\ldots (14)$$

Therefore, the endemic equilibrium point (S^*, I^*, C^*, R^*) is given by

$(S^*, I^*, C^*, R^*) =$

$$\left(\left(\frac{\mu+\sigma+\beta+b}{\alpha}\right), \left(\frac{(\mu+k)(\mu+\delta+\gamma)[\mu(\mu+\sigma+\beta+b)-\alpha n]}{\alpha\left[\frac{k[b(\mu+\delta+\gamma)+\beta\gamma]-(\mu+k)}{(\mu+\sigma+\beta+b)(\mu+\delta+\gamma)}\right]}\right), \left(\frac{\beta(\mu+k)[\mu(\mu+\sigma+\beta+b)-\alpha nN]}{\alpha[k[b(\mu+\delta+\gamma)+\beta\gamma]-(\mu+k)(\mu+\sigma+\beta+b)(\mu+\delta+\gamma)]}\right), \left(\frac{(b(\mu+\delta+\gamma)+\beta\gamma)[\mu(\mu+\sigma+\beta+b)-\alpha nN]}{\alpha[k[b(\mu+\delta+\gamma)+\beta\gamma]-(\mu+k)(\mu+\sigma+\beta+b)(\mu+\delta+\gamma)]}\right)\right) \ldots (15)$$

3. 3. Stability Analysis of Equilibrium Points for the Model

To determine the stability analysis of equilibrium point we use by Jacobian matrix. Let's consider the differential equations as the following such that:

$$\frac{dS}{dt}=n-\alpha SI-\mu S+kR=f_1(S,I,C,R) \ldots\ldots\ldots (16)$$

$$\frac{dI}{dt}=\alpha SI-(\mu+\sigma)I-\beta I-bI=f_2(S,I,C,R) \ldots\ldots\ldots (17)$$

$$\frac{dC}{dt}=\beta I-(\mu+\delta)C-\gamma C=f_3(S,I,C,R) \ldots\ldots\ldots (18)$$

$$\frac{dR}{dt}=bI+\gamma C-\mu R-kR=f_4(S,I,C,R) \ldots\ldots\ldots (19)$$

The Jacobian matrix of the dynamical system $(16)-(19)$ at any equilibrium point (S, I, C, R) is given by,

$$J(S,I,C,R) = \begin{bmatrix} \frac{\partial f_1}{\partial S} & \frac{\partial f_1}{\partial I} & \frac{\partial f_1}{\partial C} & \frac{\partial f_1}{\partial R} \\ \frac{\partial f_2}{\partial S} & \frac{\partial f_2}{\partial I} & \frac{\partial f_2}{\partial C} & \frac{\partial f_2}{\partial R} \\ \frac{\partial f_3}{\partial S} & \frac{\partial f_3}{\partial I} & \frac{\partial f_3}{\partial C} & \frac{\partial f_3}{\partial R} \\ \frac{\partial f_4}{\partial S} & \frac{\partial f_4}{\partial I} & \frac{\partial f_4}{\partial R} & \frac{\partial f_4}{\partial R} \end{bmatrix} = \begin{bmatrix} -\alpha I - \mu & -\alpha S & 0 & k \\ \alpha I & \alpha S - (\mu+\sigma+\beta+b) & 0 & 0 \\ 0 & \beta & -(\mu+\delta+\gamma) & 0 \\ 0 & b & \gamma & -(\mu+k) \end{bmatrix}$$

3. 4 Stability Analysis of Disease Free Equilibrium Point

The Jacobean matrix at disease free equilibrium points(S^0, I^0, C^0, R^0) is given as the following;

$$J\left(\frac{n}{\mu}, 0,0,0\right) = \begin{bmatrix} -\mu & \frac{-\alpha n}{\mu} & 0 & k \\ 0 & \frac{\alpha n}{\mu} - (\mu+\sigma+\beta+b) & 0 & 0 \\ 0 & \beta & -(\mu+\delta+\gamma) & 0 \\ 0 & b & \gamma & -(\mu+k) \end{bmatrix}$$

The characteristics equation is given by

$$a_4\lambda^4 + a_3\lambda^3 + a_2\lambda^2 + a_1\lambda + a_0 = 0$$

Where,

$a_4 = 1$,

$a_3 = 4\mu + \sigma + \beta + b + \delta + \gamma + k - \frac{\alpha n}{\mu}$,

$a_2 = \mu(2\mu + \delta + \gamma + k) + (\mu + k)(\mu + \delta + \gamma) - \left(\frac{\alpha n}{\mu} - (\mu + \sigma + \beta + b\right)(3\mu + \delta + \gamma + k)$,

$a_1 = \mu(\mu + k)(\mu + \delta + \gamma) - \left(\frac{\alpha n}{\mu} - (\mu + \sigma + \beta + b\right)\left(\mu(2\mu + \delta + \gamma + k) + (\mu + k)(\mu + \delta + \gamma)\right)$

$a_0 = -\mu(\mu + k)(\mu + \delta + \gamma)(\frac{\alpha n}{\mu} - (\mu + \sigma + \beta + b))$.

For the characteristics equation, $a_4\lambda^4 + a_3\lambda^3 + a_2\lambda^2 + a_1\lambda + a_0 = 0$,the Routh-Hurwitz array given by:

λ^4	1	a_2	a_0
λ^3	a_3	a_1	0
λ^2	b_1	b_2	0
λ^1	c_1	c_2	0
λ^0	d_1	0	0

Where,

$$b_1 = \frac{-1}{a_{n-1}}\begin{vmatrix} a_n & a_{n-2} \\ a_{n-1} & a_{n-3} \end{vmatrix} = \frac{a_{n-1}a_{n-2}-a_n a_{n-3}}{a_{n-1}}$$ here we do have $n = 4$ and $a_{n-5} = a_{n-6} = \cdots = 0$

$$b_1 = \frac{a_2 a_3 - a_1 a_4}{a_3} = a_2 - \frac{a_1 a_4}{a_3}$$

$$b_1 = \frac{\left[\mu(\mu+k)(\mu+\delta+\gamma) - \left(\frac{\alpha n}{\mu} - (\mu+\sigma+\beta+b\right)\left(\begin{matrix}\mu(2\mu+\delta+\gamma+k)+ \\ (\mu+k)(\mu+\delta+\gamma)\end{matrix}\right)\right]}{4\mu+\sigma+\beta+b+\delta+\gamma+k-\frac{\alpha n}{\mu}} = X^*$$

$$\mathrm{b}_2 = \frac{-1}{\mathrm{a}_{n-1}}\begin{vmatrix} \mathrm{a}_n & \mathrm{a}_{n-4} \\ \mathrm{a}_{n-1} & \mathrm{a}_{n-5} \end{vmatrix} = \frac{\mathrm{a}_{n-1}\mathrm{a}_{n-4}-\mathrm{a}_n\mathrm{a}_{n-5}}{\mathrm{a}_{n-1}} = \frac{\mathrm{a}_3\mathrm{a}_0-\mathrm{a}_4(0)}{\mathrm{a}_3} = \frac{\mathrm{a}_3\mathrm{a}_0}{\mathrm{a}_3} = \mathrm{a}_0$$

$$\Rightarrow b_2 = \mathrm{a}_0 = -\mu(\mu+k)(\mu+\delta+\gamma)\left(\frac{\alpha n}{\mu} - (\mu+\sigma+\beta+b)\right)$$

$$b_3 = \frac{-1}{a_{n-1}}\begin{vmatrix} a_n & a_{n-6} \\ a_{n-1} & a_{n-7} \end{vmatrix} = \frac{a_{n-1}a_{n-6}-a_n a_{n-7}}{a_{n-1}} = \frac{a_3(0)-a_4(0)}{a_3} = 0$$

$$c_1 = \frac{-1}{b_1}\begin{vmatrix} a_{n-1} & a_{n-3} \\ b_1 & b_2 \end{vmatrix} = \frac{b_1 a_{n-3} - a_{n-1} b_2}{b_1} = \frac{b_1 a_1 - a_3 b_2}{b_1} = a_1 - \frac{a_3 b_2}{b_1}$$

$$c_1 = a_1 - \frac{a_3 b_2}{b_1}$$

$$c_1 = \mu(\mu+k)(\mu+\delta+\gamma) - \left(\frac{\alpha n}{\mu} - (\mu+\sigma+\beta+b\right)\left(\mu(2\mu+\delta+\gamma+k) + (\mu+k)(\mu+\delta+\gamma)\right) - \frac{\left[4\mu+\sigma+\beta+b+\delta+\gamma+k-\frac{\alpha n}{\mu}\right]\left[\begin{matrix}-\mu(\mu+k)(\mu+\delta+\gamma) \\ \left(\frac{\alpha n}{\mu}-(\mu+\sigma+\beta+b)\right)\end{matrix}\right]}{X^*}$$

$$c_2 = \frac{-1}{b_1}\begin{vmatrix} a_{n-1} & a_{n-5} \\ b_1 & b_3 \end{vmatrix} = \frac{b_1 a_{n-5} - a_{n-1} b_3}{b_1} = \frac{b_1(0) - a_3(0)}{b_1} = 0$$

$$d_1 = \frac{-1}{c_1}\begin{vmatrix} b_1 & b_2 \\ c_1 & c_2 \end{vmatrix} = \frac{c_1 b_2 - c_2 b_1}{c_1} = \frac{c_1 b_2 - (0) b_1}{c_1} = \frac{c_1 b_2}{c_1} = b_2$$

$$d_1 = b_2 = -\mu(\mu+k)(\mu+\delta+\gamma)\left(\frac{\alpha n}{\mu} - (\mu+\sigma+\beta+b)\right)$$

Thus based on the Routh-Hurwitz stability criterion, the disease free equilibrium point become stable if all the values of the coefficients $a_i's$ obtained above are non-zero with the same sign and if all of the first column elements of the Routh-Hurwitz array such as a_3, b_1, c_1 *and* d_1 given above are all positive, but if at least one of the values of a_3, b_1, c_1 and d_1 is negative, the equilibrium point becomes unstable, this is because $a_4 > 0$.

3. 5. Stability Analysis of Endemic Equilibrium

The Jacobian matrix at any point (S, I, C, R) is:

$$J(S,I,C,R)=\begin{bmatrix} -\alpha I-\mu & -\alpha S & 0 & k \\ \alpha I & \alpha S-(\mu+\sigma+\beta+b) & 0 & 0 \\ 0 & \beta & -(\mu+\delta+\gamma) & 0 \\ 0 & b & \gamma & -(\mu+k) \end{bmatrix}$$

At the endemic equilibrium point the Jacobian matrix becomes

$$J(S^*,I^*,C^*,R^*)=\begin{bmatrix} -\alpha I^*-\mu & -\alpha S^* & 0 & k \\ \alpha I^* & \alpha S^*-(\mu+\sigma+\beta+b) & 0 & 0 \\ 0 & \beta & -(\mu+\delta+\gamma) & 0 \\ 0 & b & \gamma & -(\mu+k) \end{bmatrix}$$

Where $S^* = \frac{\mu+\sigma+\beta+b}{\alpha}$ *and* $\alpha S^* = \alpha\left(\frac{\mu+\sigma+\beta+b}{\alpha}\right) = \mu+\sigma+\beta+b$.

Hence, $\alpha S^* - (\mu+\sigma+\beta+b) = 0$. Hence the Jacobian matrix J becomes

$$J(S^*,I^*,C^*,R^*)=\begin{bmatrix} -\alpha I^*-\mu & -\alpha S^* & 0 & k \\ \alpha I^* & 0 & 0 & 0 \\ 0 & \beta & -(\mu+\delta+\gamma) & 0 \\ 0 & b & \gamma & -(\mu+k) \end{bmatrix}$$

Therefore, the characteristics equation becomes;

$$\Rightarrow \lambda^4 + [3\mu+\delta+\gamma+k+\alpha I^*]\lambda^3 + [(\alpha I^*+\mu)(\mu+k) + (\mu+\delta+\gamma)(\mu+\delta+\gamma) + (\alpha I^*+\mu)(\mu+\delta+\gamma) + \alpha(\mu+\sigma+\beta+b)I^*]\lambda^2 + [(\alpha I^*+\mu)(\mu+k)(\mu+\delta+\gamma) + \alpha(\mu+\sigma+\beta+b)(2\mu+\delta+\gamma+k)I^* - \alpha k I^*(\mu+\delta+\gamma)^2]\lambda + [\alpha(\mu+k)(\mu+\delta+\gamma)(\mu+\sigma+\beta+b)I^* - \alpha\beta\gamma k I^* - \alpha k I^*(\mu+\delta+\gamma)^3]$$

The characteristics equation can be written in the form of

$$a_4\lambda^4 + a_3\lambda^3 + a_2\lambda^2 + a_1\lambda + a_0 = 0.$$

Where,

$a_4 = 1,$

$a_3 = 3\mu + \delta + \gamma + k + \alpha I^*,$

$a_2 = (\alpha I^* + \mu)(\mu + k) + (\mu + \delta + \gamma)(\mu + \delta + \gamma) + (\alpha I^* + \mu)(\mu + \delta + \gamma) + \alpha(\mu + \sigma + \beta + b)I^*,$

$a_1 = (\alpha I^* + \mu)(\mu + k)(\mu + \delta + \gamma) + \alpha(\mu + \sigma + \beta + b)(2\mu + \delta + \gamma + k)I^* - \alpha k I^*(\mu + \delta + \gamma)^2,$

$a_0 = \alpha(\mu + k)(\mu + \delta + \gamma)(\mu + \sigma + \beta + b)I^* - \alpha\beta\gamma k I^* - \alpha k I^*(\mu + \delta + \gamma)^3,$

$S^* = \frac{\mu+\sigma+\beta+b}{\alpha},$

$I^* = \frac{(\mu+k)(\mu+\delta+\gamma)[\mu(\mu+\sigma+\beta+b)-\alpha n]}{\alpha[k[b(\mu+\delta+\gamma)+\beta\gamma]-(\mu+k)(\mu+\sigma+\beta+b)(\mu+\delta+\gamma)]}.$

For the characteristics equation, $a_4\lambda^4 + a_3\lambda^3 + a_2\lambda^2 + a_1\lambda + a_0 = 0$, the Routh-Hurwitz array given by:

λ^4	1	a_2	a_0
λ^3	a_3	a_1	0
λ^2	b_1	b_2	0
λ^1	c_1	c_2	0
λ^0	d_1	0	0

Where,

$b_1 = \frac{-1}{a_{n-1}}\begin{vmatrix} a_n & a_{n-2} \\ a_{n-1} & a_{n-3} \end{vmatrix} = \frac{a_{n-1}a_{n-2}-a_n a_{n-3}}{a_{n-1}}$ Since $n = 4$, we do have

$$b_1 = \frac{a_2 a_3 - a_1 a_4}{a_3} = a_2 - \frac{a_1 a_4}{a_3}$$

$$b_1 = \begin{bmatrix} (\alpha I^* + \mu)(\mu + k) + (\mu + \delta + \gamma)(\mu + \delta + \gamma) + \\ (\alpha I^* + \mu)(\mu + \delta + \gamma) + \alpha(\mu + \sigma + \beta + b)I^* \end{bmatrix} - \frac{\begin{array}{c}[(\alpha I^* + \mu)(\mu + k)(\mu + \delta + \gamma) + \\ \alpha(\mu + \sigma + \beta + b)(2\mu + \delta + \gamma + k)I^* \\ -\alpha k I^*(\mu + \delta + \gamma)^2]\end{array}}{3\mu + \delta + \gamma + k + \alpha I^*} = Y^*$$

$$b_2 = \frac{-1}{a_{n-1}}\begin{vmatrix} a_n & a_{n-4} \\ a_{n-1} & a_{n-5} \end{vmatrix} = \frac{a_{n-1}a_{n-4} - a_n a_{n-5}}{a_{n-1}} = \frac{a_3 a_0 - a_4(0)}{a_3} = a_0$$

$b_2 = a_0 = \alpha(\mu + k)(\mu + \delta + \gamma)(\mu + \sigma + \beta + b)I^* - \alpha\beta\gamma k I^* - \alpha k I^*(\mu + \delta + \gamma)^3$

$$b_3 = \frac{-1}{a_{n-1}}\begin{vmatrix} a_n & a_{n-6} \\ a_{n-1} & a_{n-7} \end{vmatrix} = \frac{a_{n-1}a_{n-6} - a_n a_{n-7}}{a_{n-1}} = \frac{a_3(0) - a_4(0)}{a_{n-1}} = 0$$

$$c_1 = \frac{-1}{b_1}\begin{vmatrix} a_{n-1} & a_{n-3} \\ b_1 & b_2 \end{vmatrix} = \frac{b_1 a_{n-3} - a_{n-1} b_2}{b_1} = \frac{b_1 a_1 - a_3 b_2}{b_1} = a_1 - \frac{a_3 b_2}{b_1}$$

$$c_1 = \begin{bmatrix} (\alpha I^* + \mu)(\mu + k)(\mu + \delta + \gamma) + \\ \alpha(\mu + \sigma + \beta + b)(2\mu + \delta + \gamma + k)I^* - \\ \alpha k I^*(\mu + \delta + \gamma)^2 \end{bmatrix} - \frac{\begin{bmatrix} \alpha(\mu+k)(\mu+\delta+\gamma)(\mu+\sigma+\beta+b)I^* - \\ \alpha\beta\gamma k I^* - \alpha k I^*(\mu+\delta+\gamma)^3 \end{bmatrix} [3\mu+\delta+\gamma+k+\alpha I^*]}{Y^*}$$

Where,

$$X^* = b_1$$

$$c_2 = \frac{-1}{b_1}\begin{vmatrix} a_{n-1} & a_{n-5} \\ b_1 & b_3 \end{vmatrix} = \frac{b_1 a_{n-5} - a_{n-1} b_3}{b_1} = \frac{b_1(0) - a_3 b_3}{b_1} = \frac{a_3 b_3}{b_1} = 0$$

$$c_2 = 0$$

$$d_1 = \frac{-1}{c_1}\begin{vmatrix} b_1 & b_2 \\ c_1 & c_2 \end{vmatrix} = \frac{c_1 b_2 - c_2 b_1}{c_1} = b_2 - \frac{c_2 b_1}{c_1} = b_2 - \frac{(0) b_1}{c_1} = b_2$$

$$d_1 = b_2 = a_0 = \alpha(\mu + k)(\mu + \delta + \gamma)(\mu + \sigma + \beta + b)I^* - \alpha\beta\gamma k I^* - \alpha k I^*(\mu + \delta + \gamma)^3$$

Therefore, by the Routh stability criterion[8] if the values of all the coefficients a_3, a_2, a_1 *and* a_0 obtained above are non-zero with the same algebraic sign and if all the first column elements a_3, b_1, c_1 *and* d_1 are positive, then the endemic equilibrium point becomes stable otherwise it is unstable.

3.6. Reproduction Number of the Model

We find the reproduction number by using the next generation method such that the reproduction number is the dominant Eigen value of the next generation matrix $G = FV^{-1}$ where F represents new infection and V represent transfers of infections from one compartment to another[9]. Now to find the reproduction number R_0 by using the next generation method, we can rearrange the differential equations of the dynamical system (5) – (8) as follows;

$$\frac{dI}{dt} = \alpha SI - (\mu + \sigma)I - \beta I - bI \ldots\ldots\ldots\ldots\ldots\ldots\ldots\ldots\ldots\ldots (20)$$

$$\frac{dC}{dt} = \beta I - (\mu + \delta)C - \gamma C \ldots\ldots (21)$$

$$\frac{dS}{dt} = n - \alpha SI - \mu S + kR \ldots\ldots (22)$$

$$\frac{dR}{dt} = bI + \gamma C - \mu R - kR \ldots\ldots (23)$$

The basic matrix of the second generation number is:

$$f(I,C,S,R) = \begin{pmatrix} \alpha SI \\ 0 \\ 0 \\ 0 \end{pmatrix} \text{ and } v(I,C,S,R) = \begin{pmatrix} (\mu+\sigma+\beta+b)I \\ (\mu+\delta+\gamma)C - \beta I \\ \mu S - (n+kR) \\ (\mu+k)R - (bI+\gamma C) \end{pmatrix}$$

So $$V^{-1} = \begin{bmatrix} \frac{1}{\mu+\sigma+\beta+b} & 0 \\ \frac{\beta}{(\mu+\sigma+\beta+b)(\mu+\delta+\gamma)} & \frac{1}{\mu+\delta+\gamma} \end{bmatrix}$$

$$FV^{-1} = \begin{bmatrix} \frac{\alpha n}{\mu(\mu+\sigma+\beta+b)} & 0 \\ 0 & 0 \end{bmatrix}$$

The characteristics polynomial equation is $\begin{vmatrix} \frac{\alpha n}{\mu(\mu+\sigma+\beta+b)} - \lambda & 0 \\ 0 & 0 - \lambda \end{vmatrix} = 0$

Therefore the required reproduction number R_0 is ; $R_0 = \rho(FV^{-1}) = \frac{\alpha n}{\mu(\mu+\sigma+\beta+b)}$

Hence, $R_0 = \frac{\alpha n}{\mu(\mu+\sigma+\beta+b)} \ldots\ldots (24)$

4. Real Parameter Estimation

To study the spread of typhoid fever in Sheno town by using the modeling mechanisms of infectious diseases, we adopt the SICRS compartmental model by classifying the total population N as S: Susceptible, I: Infectious, C: Carrier, and R: Recovered.

The assumption that we considered in the parameter estimation of the model are:

- The disease is transmitted in a closed environment, meaning that the total population of Sheno town has a constant size N.

- The population is heterogeneously mixing with no restriction of age, sex, occupation and religion.
- The disease is transmitted by direct transmission or by ingestion of contaminated food or liquid by susceptible individuals.
- All new born are susceptible and they inter in to susceptible group by net birth rate n.
- The natural death rate in all compartments is considered as equal.
- Successfully treated individual can become infected again.
- The typhoid induced death rate and carrier induced death rate are equal.

We estimate the parameters of the model based on the data that collected from Sheno town in the year $01/02/2009 - 05/07/2010$ E.C and other sources which can help us to parameterize our model as follows;

Description	Values
Number of total population	15891
Number of initial infected population	1421
Number of initial carriers population	381
Number of initial recovered population	1369
Number of new born in Sheno town	1413
Number of people who again become susceptible after recovered	1040
Total number of natural death in Sheno town	451

Table 2 : The gathered data about the total population and typhoid fever in sheno town.

Classes of the initial populations	Symbol	Total number
Susceptible	S_0	14470
Infected	I_0	1421
Carrier	C_0	381
Recovered	R_0	1369

Table 3:-The total number of initial populations in each of the compartments of the model.

According to [1] Per capital recovery rate of infective (b) is taken as the inverse of time between typhoid fever infection and recovery by treatment. This within 4 and 6 weeks. Take the treatment to be 5 weeks (35 days). Therefore recovery rate of infective becomes;

$$b = \frac{1}{35} = 0.0286$$

The recovery rate of carriers γ is the inverse of time between carrier state and recovery by continuous treatment. According to [1] it is 6 weeks and beyond implying that $\gamma < 1$ per year. Taken the treatment to be 42 a days. Therefore recovery rate if infective becomes;

$$\gamma = \frac{1}{42} = 0.0238$$

Per capita progression rate from- infective to carrier β: This range from month to decades. Most infective never progress towards the carrier state. However, average progression is short and become shorter due to the availability of vaccines now.it is about three to five percent (3-5%) of typhoid fever patients become carriers. Thus the average range of the progression become, $\frac{(30+3650)}{2} days =$ 1840 days. Therefore the progression rate becomes;

$$\beta = \frac{1}{1840} = 0.0005,$$

$$n = \frac{\text{Number of new born individual}}{\text{Total number of population}} = \frac{1413}{15891} = 0.0889,$$

$$\mu = \frac{\text{Number of total natural death}}{\text{Total population}} = \frac{451}{15891} = 0.0284,$$

$$\alpha = \frac{\text{Number of typhoid fever infectious}}{\text{Total population}} = \frac{1421}{15891} = 0.0894,$$

$$k = \frac{\text{Number of people who again into the susecptible class}}{\text{number of the recoverde people}} = \frac{1040}{1369} = 0.7597.$$

Descriptions	Symbols	Values	Source
The recruitment rate	n	0.0889	Estimated
The natural death rate	μ	0.0284	Estimated
The rate of infection	α	0.0894	Estimated
The rate of recovery from the infectious stage	b	0.0286	[1]
The typhoid fever-induced death rate	σ	0.0006	[10]

The rate of progression from infective to carrier	β	0.0005	[1]
The carrier-induced death rate	δ	0.0006	[10]
The rate of recovery from carrier stage	γ	0.0238	[1]
The rate of re-susceptible	k	0.7597	Estimated

Table 4:- The table contains the calculated parameters.

From equation (24) the reproduction number is given by,

$$R_0 = \frac{\alpha n}{\mu(\mu+\sigma+\beta+b)},$$

where $\alpha = 0.0894, n = 0.0889, \mu = 0.0284, \sigma = 0.0006, \beta = 0.0005,\ b = 0.0286$.

$$\Rightarrow R_0 = \frac{0.0079}{0.0016} = 4.9375.$$

5. Sensitivity Analysis

Through the process of sensitivity indices of the effective reproduction number of the spread and control of typhoid fever model to all relevant parameters, we have shown how crucial each parameter is to the propagation of the disease in this section. The magnitude of the sensitivity index for the parameter that is the most sensitive is greater than that for all other parameters [11,12]. For R_0, the sensitivity analyses have been conducted is presented with the following Figure below.

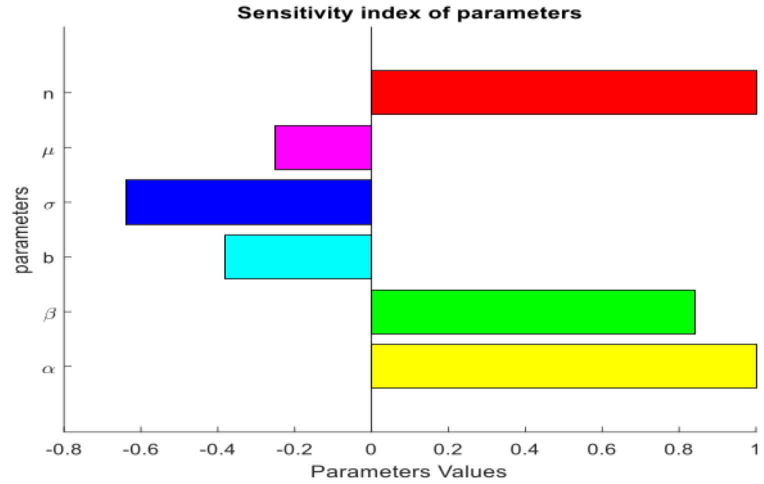

Figure.3 Sensitivity Analysis

The parameter values from sensitivity analysis of Fig.3 provide further insights into the dynamics of typhoid fever in Sheno Town, Ethiopia. The rate of infection (α) and recruitment rate (n), both set to 1, indicate a high potential for disease transmission and population growth. The negative values for natural death rate (μ), typhoid fever-induced death rate (σ), and the rate of recovery from the infectious stage (b) suggest potential decreases in overall mortality, typhoid-related mortality, and recovery from the infectious stage, respectively.

The positive value for the rate of progression from infective to carrier (β) at 0.84 suggests an increase in the transition from infective to carrier stage, possibly influencing disease persistence. These adjustments highlight the importance of understanding the interplay between different parameters in shaping the dynamics of typhoid fever.

In interpreting these results, it becomes crucial to reevaluate intervention strategies. Strategies aimed at reducing the rate of infection, enhancing recovery from the infectious stage, and addressing the progression to carrier stage may play a pivotal role in controlling typhoid fever in Sheno Town. Moreover, a comprehensive approach considering the impact on mortality rates and the potential for population growth is essential for effective public health planning and implementation. Regular monitoring and adaptability of strategies based on ongoing evaluations will be essential for dynamic and successful disease management.

6. Numerical Simulation

In the context of the SICRS model for typhoid fever, the local asymptotic stability of the disease-free equilibrium point signifies that, under certain conditions, the system converges towards a state where no individuals in the population are infected. A visual demonstration of this concept has been shown in Fig. 4 below, where each axis represents a different compartment of the model.

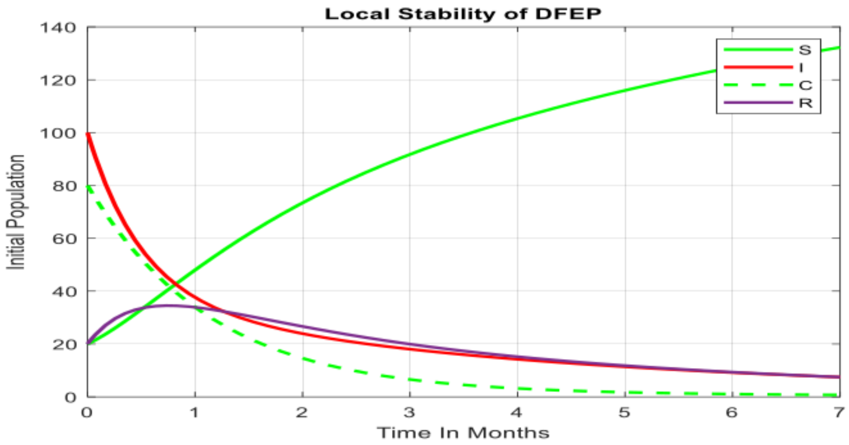

Figure 5. Local Stability of DFE

This graphical representation, which is given as Fig.5 above, offers an intuitive understanding of the system's behavior near the disease-free state and provides a valuable insight into the stability properties of the mathematical model.

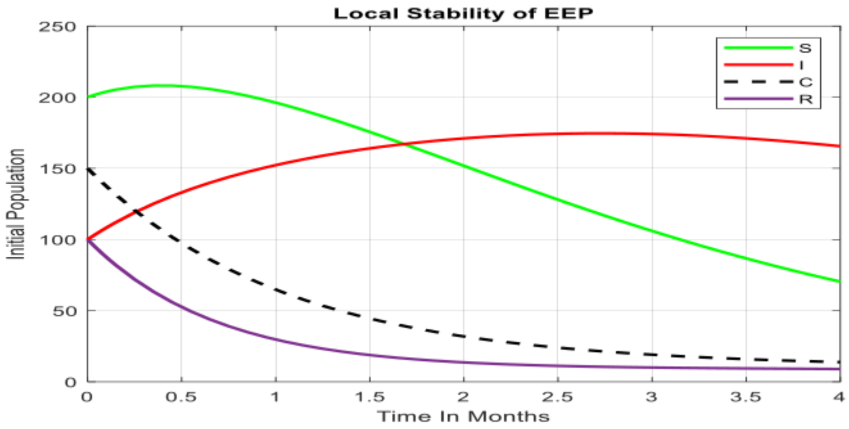

Figure 6. Local Stability of EEP

The local stability of endemic equilibrium points is crucial for understanding the persistent presence of the disease within a population. This graphical representation, along with an optional eigenvalue analysis, provides a comprehensive understanding of the long-term dynamics of the disease, shedding light on whether typhoid fever is likely to persist or be controlled under specific conditions in the mathematical model.

Moreover, we consider the reproduction number of the extended SICRS mathematical model, $R_0 = \frac{\alpha n}{\mu(\mu+\sigma+\beta+b)}$, which depends on six parameters: namely the rate of infection(α), recruitment rate(n), natural death rate(μ), typhoid induced death rate(σ), the rate progression from infective to carrier(β), the rate of recovery from the infectious stage(b). In this analysis we discuss the effect of each parameters change on the reproduction number graphically using win plot software, where the parameters values are taken from table 4, that is $\alpha = 0.0894$, $n = 0.0889$, $\mu = 0.0284, \sigma = 0.0006, \beta = 0.0005, b = 0.0286$.

1. Let we take our control parameter α, the rate of infection and the remaining parameters are constant. That is, take $n = 0.0889, \mu = 0.0284, \sigma = 0.0006, \beta = 0.0005, b = 0.0286$.

Therefore we do have $R_0 = \left(\frac{n}{\mu(\mu+\sigma+\beta+b)}\right)\alpha$

$$\Rightarrow R_0 = \left(\frac{0.0889}{0.0284(0.0284+0.0006+0.0005+0.0286)}\right)\alpha = \left(\frac{(0.0889)}{0.0284(0.0581)}\right)\alpha$$

$$\Rightarrow R_0 = 55.5625\alpha$$

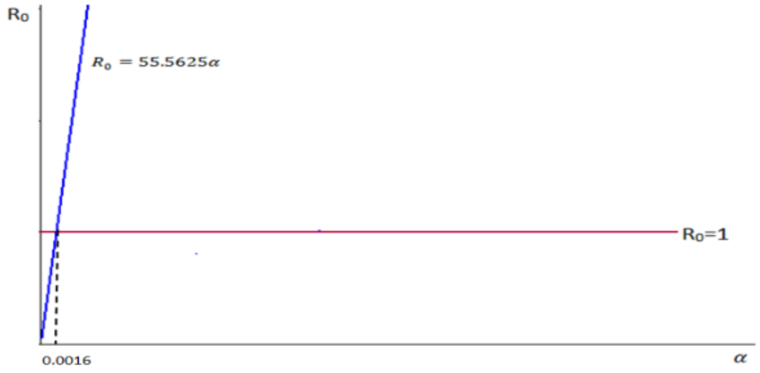

Figure -7 Reproduction number versus the rate of infection.

We observe from the graph in Fig.7, that the reproduction number and the rate of infection are proportional.

2. Let we take our control parameter n, recruitment rate and the remaining parameters are constant. That is, take $\alpha = 0.0894, \mu = 0.0284$, $\sigma = 0.0006$, $\beta = 0.0005, b = 0.0286$.

Therefore we do have $R_0 = \left(\frac{\alpha}{\mu(\mu+\sigma+\beta+b)}\right)n$

$$\Rightarrow R_0 = \left(\frac{0.0894}{0.0284(0.0284+0.0006+0.0005+0.0286)}\right)n = \left(\frac{0.0894}{0.0284(0.0581)}\right)n$$

$$\Rightarrow R_0 = 55.875n$$

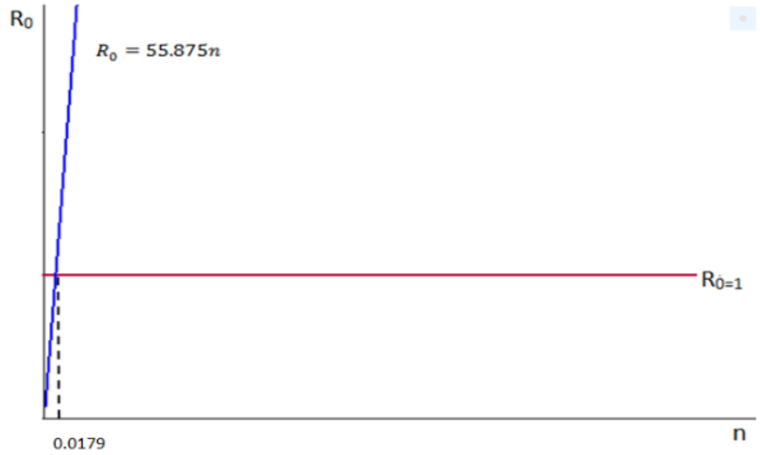

Figure -8 Reproduction number versus recruitment rate.

We observe from the Fig.8 that the reproduction number and the recruitment rate are proportional.

3. Let we take our control parameter μ , natural death rate and the remaining parameters are constant. That is, take $n = 0.0889, \alpha = 0.0894$, $\sigma = 0.0006, \beta = 0.0005, b = 0.0286$.

Therefore $R_0 = \frac{\alpha n}{\mu(\mu+\sigma+\beta+b)} = \frac{(0.0894)(0.0889)}{\mu(\mu+0.0006+0.0005+0.0286)} = \frac{0.0079}{\mu(\mu+0.0297)} = \frac{0.0079}{\mu(\mu+0.0297)}$

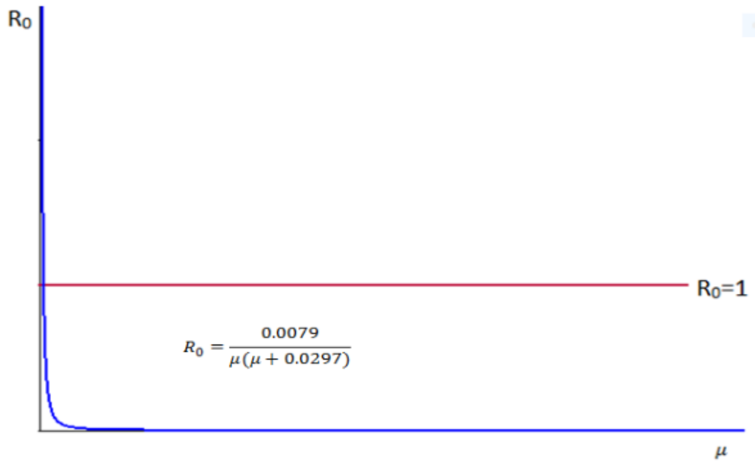

Figure 9 Reproduction number versus natural death rate.

We observe from the Fig. 9 that the reproduction number and the natural death rate are inversely proportional.

4. Let we take our control parameter σ, the typhoid induced death rate and the remaining parameters are constant. That is, take $\alpha = 0.0894$, $n = 0.0889$, $\mu = 0.0284$ $\beta = 0.0005$, $b = 0.0286$.

Therefore we do have $R_0 = \frac{(0.0894)(0.0889)}{0.0284(0.0284+\sigma+0.0005+0.0286)} = \frac{0.0079}{0.0284(\sigma+0.0575)}$

$$\Rightarrow R_0 = \frac{0.0079}{0.0284\sigma + 0.0016}$$

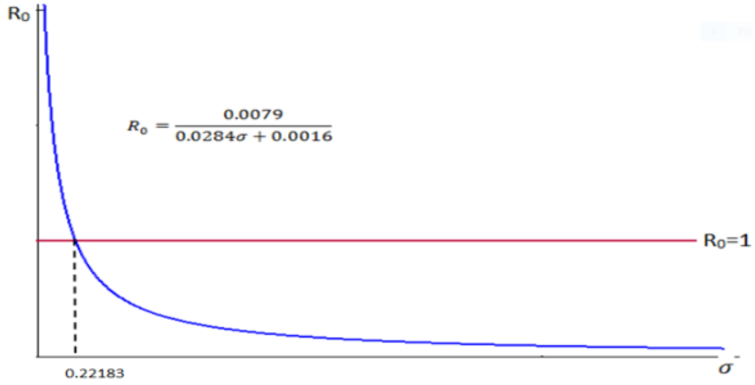

Figure 10: Reproduction number versus typhoid induced death rate.

We observe from Fig.10 that the reproduction number and the typhoid induced death rate are inversely proportional.

5. Let we take our control parameter β, the rate of progression from infective to carrier and the remaining parameters are constant. That is, take $\alpha = 0.0894$, $n = 0.0889$, $\mu = 0.0284$, $\sigma = 0.0006$, $b = 0.0286$.

Therefore we do have $R_0 = \frac{(0.0894)(0.0889)}{0.0284(0.0284+0.0006+\beta+0.0286)} = \frac{0.0079}{0.0284(\beta+0.0576)}$

$$\Rightarrow R_0 = \frac{0.0079}{0.0284\beta + 0.0016}$$

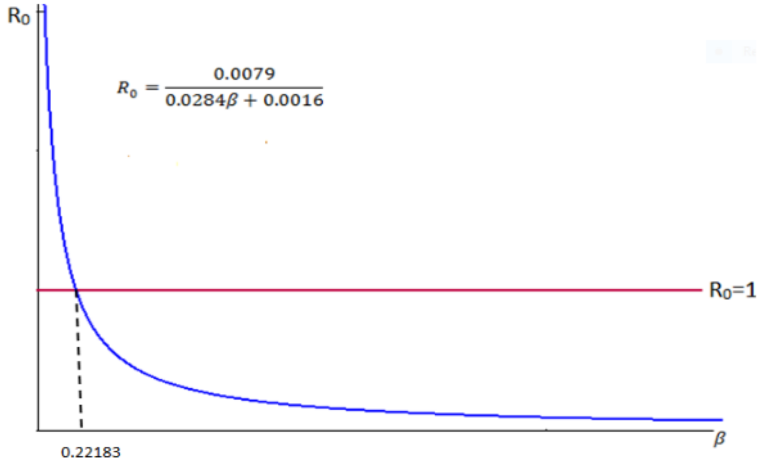

Figure 11 Reproduction number versus rate of progression from infective to carrier.

We observe from the Fig.11 that the reproduction number and the rate of progression from infective to carrier are inversely proportional.

6. Let we take our control parameter b, the rate of recovery from infectious stage and the remaining parameters are constant. That is, take $\alpha = 0.0894$, $n = 0.0889$, $\mu = 0.0284$, $\sigma = 0.0006$, $\beta = 0.0005$.

Therefore we do have $R_0 = \frac{(0.0894)(0.0889)}{0.0284(0.0284+0.0006+0.0005+b)} = \frac{0.0079}{0.0284(b+0.0295)} = \frac{0.0079}{0.0284b+0.0008}$

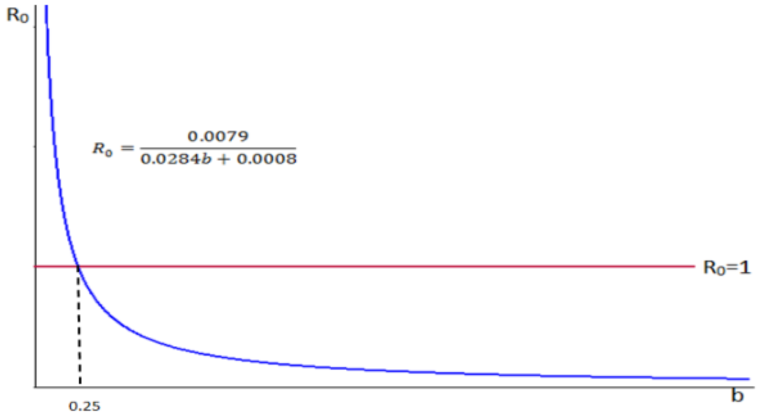

Figure 12 Reproduction number versus rate of recovery from the infectious stage.

We observe from Fig.12 that the reproduction number and the rate of recovery from infectious stage are inversely proportional.

7. Result and Discussion

The SICRS model for typhoid fever, formulated through a deterministic system of differential equations, serves as a powerful tool for predicting and understanding the dynamics of disease transmission. In this study, we conducted a comprehensive qualitative analysis, ensuring the epidemiological and mathematical soundness of the model by establishing the positivity and boundedness of solutions. The model revealed two critical equilibrium points: the disease-free equilibrium and the endemic equilibrium. To assess the potential spread of typhoid fever in Sheno town, we introduced the basic reproduction number (R_0), a pivotal metric in epidemiology. Utilizing real data, our extended model yielded an R_0 of 4.9375, indicating a concerning level of disease transmission.

To delve into the implications of our findings, we employed numerical simulations and stability analyses. Routh-Hurwitz stability criteria provided insights into the stability of equilibrium points, revealing the disease-free equilibrium as unstable and the endemic equilibrium as stable. Our graphical representations vividly illustrate the impact of varying parameters on R_0 and disease spread. Notably, increased infection rates and recruitment rates correlated with higher R_0 values, signifying heightened disease transmission. Conversely, lower values of these parameters resulted in decreased R_0 , indicating a reduction in the spread of typhoid fever.

Noteworthy observations emerged from our simulations. We identified that changes in the rate of infection and recruitment significantly influenced R_0 , demonstrating their pivotal roles in disease dynamics. Additionally, the typhoid-induced death rate, progression rate, and rate of recovery from the infective stage exhibited clear impacts on R_0 , offering valuable insights for targeted interventions. Importantly, our results underscored the effectiveness of reducing infection and recruitment rates in controlling the transmission of typhoid fever.

In conclusion, our study combines mathematical rigor with real-world applicability to provide a nuanced understanding of typhoid fever dynamics in Sheno town. The graphical representations not only enhance the interpretability of our results but also serve as powerful tools for communicating the implications of various interventions. This research contributes valuable insights for public health strategies, emphasizing the significance of targeted measures to curb infection and recruitment rates for effective typhoid fever control in Sheno town and similar settings.

8. Conclusion and Recommendation

9. Conclusion

In this paper, a non-linear mathematical model was formulated and then we have seen their stability analysis. From the stability analysis and numerical simulation result, the researcher concluded that the disease spread through the community of Sheno town without control measure and the number of infective individuals increases in the community. Since the reproduction number $R_0 = 4.9375$ which is greater than one.

9.1 Recommendation

To reduce the spread of typhoid fever in Sheno town we recommended the following suggestion.

1. Control the parametric values of infectious rate $\alpha < 0.0016$, by fixing the remaining parametric values.
2. Control the parametric values of recruitment rate $n < 0.0179$, by fixing the remaining parametric values.

In general when these two control parametric value $\alpha<0.0016$ and $n<0.0179$, the SICRS model reproduction number less than one, then the transmission of disease decrease and the disease will be controlled.

10. Limitation of the Research

In this thesis to collect data we considered only Kebeles of Sheno town without including the nearby Kebeles which can get health service at Sheno town health institution. Also we considered all new born was healthy themselves and they enter in to susceptible class directly and we collected data by ignoring the immigration and migration of population into and out of Sheno town respectively. Thus we suggest that by giving attention for this limitation and making improvements on them, the future work will be better on controlling the spread of typhoid fever.

11. Reference

1. Scholarship W, Ashrafur Rahman S, ZOu X. *Study of Infectious Diseases by Mathematical Models: Study of Infectious Diseases by Mathematical Models: Predictions and Controls Predictions and Controls*.; 2016. https://ir.lib.uwo.ca/etd://ir.lib.uwo.ca/etd/3487

2. Cvj B, Dixoy~ H. *19-74A*. *Epidemiological and Statistical Methodology Unit*. *World Health Organization*. Vol 7.

3. Teklu SW, Kotola BS. A dynamical analysis and numerical simulation of COVID-19 and HIV/AIDS co-infection with intervention strategies. *J Biol Dyn*. 2023;17(1). doi:10.1080/17513758.2023.2175920

4. Höhle M. *Infectious Disease Modelling*.; 2015. http://www.math.su.se/~hoehle/pubs/Hoehle_

5. Kotola BS, Teklu SW. A Mathematical Modeling Analysis of Racism and Corruption Codynamics with Numerical Simulation as Infectious Diseases. *Comput Math Methods Med*. 2022;2022. doi:10.1155/2022/9977727

6. Beyene G, Asrat D, Mengistu Y, Aseffa A, Wain J. *Typhoid Fever in Ethiopia*.; 2008.

7. Simon PL, Taylor M, Kiss IZ. Exact epidemic models on graphs using graph-automorphism driven lumping. *J Math Biol*. 2011;62(4):479-508. doi:10.1007/s00285-010-0344-x

8. Edward S. Modelling and Stability Analysis of Typhoid Fever Transmission Dynamics with control Strategies. *International Journal of Sciences: Basic and Applied Research (IJSBAR) International Journal of Sciences: Basic and Applied Research*. 2017;32(1):151-168. http://gssrr.org/index.php?journal=JournalOfBasicAndApplied

9. Van Den Driessche P, Watmough J. *Reproduction Numbers and Sub-Threshold Endemic Equilibria for Compartmental Models of Disease Transmission*. www.elsevier.com/locate/mbs

10. Crump JA, Luby SP, Mintz ED. *The Global Burden of Typhoid Fever*. Vol 82.; 2004.

11. Kotola BS, Gebru DM, Alemneh HT. Appraisal and Simulation on Codynamics of Pneumonia and Meningitis with Vaccination Intervention: From a Mathematical Model Perspective. *Comput Math Methods Med*. 2022;2022. doi:10.1155/2022/9945047

12. kotola BS, Mekonnen TT. Mathematical model analysis and numerical simulation for codynamics of meningitis and pneumonia infection with intervention. *Sci Rep*. 2022;12(1). doi:10.1038/s41598-022-06253-0

makinde

ORIGINALITY REPORT

37%	33%	23%	23%
SIMILARITY INDEX	INTERNET SOURCES	PUBLICATIONS	STUDENT PAPERS

PRIMARY SOURCES

1	Submitted to Haramaya University<br>Student Paper	13%
2	etd.dbu.edu.et<br>Internet Source	6%
3	www.jidc.org<br>Internet Source	3%
4	www.oer.unn.edu.ng<br>Internet Source	2%
5	mafiadoc.com<br>Internet Source	2%
6	opastpublishers.com<br>Internet Source	1%
7	Submitted to Wollega University<br>Student Paper	1%
8	Submitted to Federal University of Technology<br>Student Paper	1%
9	projecteuclid.org<br>Internet Source	1%

10	Lecture Notes in Mathematics, 2008.<br>Publication	1 %
11	Vicente Alonso Navarro Valencia, Yamilka Díaz, Juan Miguel Pascale, Maciej F. Boni, Javier E. Sanchez-Galan. "Using compartmental models and Particle Swarm Optimization to assess Dengue basic reproduction number R0 for the Republic of Panama in the 1999-2022 period", Heliyon, 2023<br>Publication	1 %
12	Submitted to Texas Christian University<br>Student Paper	1 %
13	Christopher Saaha Bornaa, Baba Seidu, Yakubu Ibrahim Seini. "Modeling the impact of early interventions on the transmission dynamics of coronavirus infection", F1000Research, 2021<br>Publication	<1 %
14	Submitted to George Bush High School<br>Student Paper	<1 %
15	Submitted to University of Lincoln<br>Student Paper	<1 %
16	medtextfree.wordpress.com<br>Internet Source	<1 %

17	Firaol Asfaw Wodajo, Temesgen Tibebu Mekonnen. "Mathematical model analysis and numerical simulation of intervention strategies to reduce transmission and re-activation of hepatitis B disease", F1000Research, 2022<br>Publication	<1 %
18	pdfs.semanticscholar.org<br>Internet Source	<1 %
19	wojast.com<br>Internet Source	<1 %
20	Hamadjam Abboubakar, Reinhard Racke. "Mathematical modeling, forecasting, and optimal control of typhoid fever transmission dynamics", Chaos, Solitons & Fractals, 2021<br>Publication	<1 %
21	ugspace.ug.edu.gh<br>Internet Source	<1 %
22	Submitted to Somerville House<br>Student Paper	<1 %
23	Tsegaye Kebede Irena, Sunita Gakkhar. "A dynamical model for HIV-typhoid co-infection with typhoid vaccine", Journal of Applied Mathematics and Computing, 2021<br>Publication	<1 %
24	www.ctresources.info<br>Internet Source	

<1%

25 Lei Wang, Zhidong Teng, Long Zhang. "Global Behaviors of a Class of Discrete SIRS Epidemic Models with Nonlinear Incidence Rate", Abstract and Applied Analysis, 2014 <1%
Publication

26 Submitted to University of Glamorgan <1%
Student Paper

27 Submitted to Washington High School <1%
Student Paper

28 wallaby.vu.edu.au <1%
Internet Source

29 www.medicinenet.com <1%
Internet Source

30 Shewafera Wondimagegnhu Teklu, Belela Samuel Kotola. "A dynamical analysis and numerical simulation of COVID-19 and HIV/AIDS co-infection with intervention strategies", Journal of Biological Dynamics, 2023 <1%
Publication

31 W. Edwin Harris. "THE MAINTENANCE OF HERITABLE VARIATION THROUGH SOCIAL COMPETITION", Evolution, 2/2008 <1%
Publication

32	Submitted to The African Institute for Mathematical Sciences<br>Student Paper	<1%
33	pubmed.ncbi.nlm.nih.gov<br>Internet Source	<1%
34	www.medrxiv.org<br>Internet Source	<1%
35	Attaullah, Şuayip Yüzbaşı, Sultan Alyobi, Mansour F. Yassen, Wajaree Weera. "A Higher-Order Galerkin Time Discretization and Numerical Comparisons for Two Models of HIV Infection", Computational and Mathematical Methods in Medicine, 2022<br>Publication	<1%
36	F. E. Laine-Pearson. "Instabilities of multiphase wave trains in coupled nonlinear Schrödinger equations: A bisymplectic framework", Journal of Mathematical Physics, 2005<br>Publication	<1%
37	repository-tnmgrmu.ac.in<br>Internet Source	<1%
38	Engida Endriyas Endashaw, Temesgen Tibebu Mekonnen. "Modeling the Effect of Vaccination and Treatment on the Transmission Dynamics of Hepatitis B Virus	<1%

and HIV/AIDS Coinfection", Journal of Applied Mathematics, 2022
Publication

39	Lunga Matsebula, Farai Nyabadza. "Mathematical analysis of cholera typhoid co-infection transmission dynamics", Frontiers in Applied Mathematics and Statistics, 2022<br>Publication	<1 %
40	Muhammad Sinan, Kamal Shah, Poom Kumam, Ibrahim Mahariq, Khursheed J. Ansari, Zubair Ahmad, Zahir Shah. "Fractional order mathematical modelling of typhoid fever disease", Results in Physics, 2021<br>Publication	<1 %
41	Schuette, M.C.. "A qualitative analysis of a model for the transmission of varicella-zoster virus", Mathematical Biosciences, 200304<br>Publication	<1 %
42	Shewafera Wondimagegnhu Teklu, Birhanu Baye Terefe, Dejen Ketema Mamo, Yohannes Fissha Abebaw. "Optimal control strategies on HIV/AIDS and pneumonia co-infection with mathematical modelling approach", Journal of Biological Dynamics, 2023<br>Publication	<1 %
43	ds.amu.edu.et<br>Internet Source	<1 %

44	uilis.unsyiah.ac.id<br>Internet Source	<1 %
45	www.researchgate.net<br>Internet Source	<1 %
46	Belela Samuel Kotola, Dawit Melese Gebru, Haileyesus Tessema Alemneh. "Appraisal and Simulation on Codynamics of Pneumonia and Meningitis with Vaccination Intervention: From a Mathematical Model Perspective", Computational and Mathematical Methods in Medicine, 2022<br>Publication	<1 %
47	Belela Samuel Kotola, Shewafera Wondimagegnhu Teklu. "A Mathematical Modeling Analysis of Racism and Corruption Codynamics with Numerical Simulation as Infectious Diseases", Computational and Mathematical Methods in Medicine, 2022<br>Publication	<1 %
48	Magombedze, G.. "Mathematical modelling of immune regulation of type 1 diabetes", BioSystems, 201011/12<br>Publication	<1 %
49	Shewafera Wondimagegnhu Teklu, Temesgen Tibebu Mekonnen. "HIV/AIDS-Pneumonia Coinfection Model with Treatment at Each Infection Stage: Mathematical Analysis and	<1 %

Numerical Simulation", Journal of Applied Mathematics, 2021
Publication

Exclude quotes Off
Exclude bibliography On
Exclude matches Off
