# Supplementary material for: Dynamics and control of typhoid fever in Sheno town, Ethiopia: A comprehensive nonlinear model for transmission analysis and effective intervention strategies
Source: PLoS One. 2024 Aug 7;19(8):e0306544. doi: 10.1371/journal.pone.0306544 (PMC11305579; doi:10.1371/journal.pone.0306544)
Supplement: Attachment — Submitted filename: Review.docx [file pone.0306544.s002.docx]

Dear Prof,

I am sincerely grateful for this opportunity to review for this journal but the plagiarism is on the high side 37%, I will advise the author to reduce the similarity index before proceeding to the reviewing process.

Attached is the result of a similarity test.

Thank you.

John Akanni, Ph.D
