# Supplementary material for: Dynamics and control of typhoid fever in Sheno town, Ethiopia: A comprehensive nonlinear model for transmission analysis and effective intervention strategies
Source: PLoS One. 2024 Aug 7;19(8):e0306544. doi: 10.1371/journal.pone.0306544 (PMC11305579; doi:10.1371/journal.pone.0306544)
Supplement: Attachment — Submitted filename: Response to Reviewer.docx [file pone.0306544.s003.docx]

Dear Prof. Oluwole Daniel Makinde

I am writing to extend my sincere gratitude for your continued guidance throughout the review process of our manuscript titled "Dynamics and Control of Typhoid Fever in Sheno Town: A Comprehensive Nonlinear Model for Transmission Analysis and Effective Intervention Strategies," bearing the identification number PONE-D-23-42909.

I am pleased to inform you that we have meticulously revised the manuscript in response to the invaluable feedback provided by the reviewers. Enclosed, you will find the updated version of our manuscript along with a detailed document addressing each of the reviewers' comments.

We greatly appreciate the time and effort invested by the reviewers in critically evaluating our work. Their insights have been instrumental in enhancing the quality and clarity of our manuscript. We are deeply committed to upholding the rigorous standards of academic scholarship, and we believe that the revisions made will further strengthen the contribution of our research to the scientific community.

Once again, we extend our gratitude to you and the reviewers for your invaluable contributions to this process. We eagerly anticipate your feedback on the revised manuscript and remain at your disposal for any further guidance or clarification you may require.

Thank you for your unwavering support and consideration.

Warm regards,

Belela Samuel (PhD)

**Reviewer #1:**

I am sincerely grateful for this opportunity to review for this journal but the plagiarism is on the high side 37%, I will advise the author to reduce the similarity index before proceeding to the reviewing process.

**Response to Reviewer #1:**

We extend our sincere gratitude for your conscientious review of our manuscript. Your dedication to upholding academic standards and ensuring the integrity of scholarly work is deeply appreciated. We are pleased to inform you that we have taken immediate action to address the issue of plagiarism, diligently working to reduce the similarity index in accordance with your feedback.

Your meticulous assessment has undoubtedly contributed to the refinement of our work, and we are thankful for your valuable insights. Your commitment to scholarly rigor is commendable, and we are honored to have benefited from your expertise in this process.

Once again, we express our gratitude for your time and efforts in reviewing our manuscript. Should you have any further comments or suggestions, please do not hesitate to share them with us. Your continued engagement is invaluable to us as we strive to uphold the highest standards of academic excellence.

Warm regards,

Belela Samuel(PhD)

**Reviewer #2:**

The introduction should be enriched.
The references are not uniformly written
The author should consider writing the mathematical equations as a mathematician.
There are so many grammatical and typo errors, the author should consider correcting it
The author may consider improving the writeup with the following relevant papers: 
----------Modelling the Super-infection of Two Strains of Dengue Virus Journal of the Egyptian Mathematical
Society 2023, 31:1 DOI:<https://doi.org/10.1186/s42787-023-00161-6>
-----------Mathematical Analysis of Two Strains of COVID-19 Using SEIR Model J. Math. Fund. Sci. Vol. 54, No.2, 2022, 211-232.
-----------Impact of Corruption in a Society with Exposed Honest Individuals: A Mathematical Model Asia Pac. J. Math. 2023 10:18
-----------Analysis of COVID-19 disease with Careless Infective using SEITRS model Asia Pac. J. Math. 2023 10:10

**Response to Reviewer #2:**

We extend our gratitude for your meticulous evaluation of our manuscript and for providing valuable suggestions for improvement. We are pleased to inform you that we have already taken proactive measures to address the issues raised:

**Enrichment of Introduction:** The introduction has been revised and enriched to provide a more comprehensive overview of the topic, ensuring that it effectively contextualizes our study.

**Uniformity in References:** All references have been uniformly formatted according to the prescribed style guidelines of the journal.

**Mathematical Equations:** The mathematical equations have been revised to meet the standards expected of mathematicians, ensuring clarity and precision in their presentation.

**Grammatical and Typo Errors:** We have conducted a thorough proofreading and editing process to rectify any grammatical and typographical errors present in the manuscript, ensuring that the text is error-free.

**Incorporation of Relevant Papers:** The suggested relevant papers have been reviewed, and relevant insights and findings have been incorporated into our manuscript to enrich the discussion and strengthen our analysis.

We sincerely appreciate your attention to detail and your commitment to maintaining the scholarly integrity of our work. Your feedback has been invaluable in guiding us towards enhancing the quality and impact of our research.

Finally, your continued engagement is highly valued, and we are grateful for the opportunity to incorporate your expertise into our work.

Warm regards,

Belela Samuel (PhD)
