# Supplementary material for: Dynamics and control of typhoid fever in Sheno town, Ethiopia: A comprehensive nonlinear model for transmission analysis and effective intervention strategies
Source: PLoS One. 2024 Aug 7;19(8):e0306544. doi: 10.1371/journal.pone.0306544 (PMC11305579; doi:10.1371/journal.pone.0306544)
Supplement: Attachment — Submitted filename: PLOS.docx [file pone.0306544.s004.docx]

Page 13 check the characteristics equation again,

Page 14 check the Ro again.

The Sensitivity analysis is it local or global ?

I do not think Figure 3 is correct, please check.

The write up needs total repacking, the concept is okay, method is right and the results looks meaningful but poor write up .

The article is not properly written, it is just cope and paste of his/her thesis, please work on the grammars and tense.

For example; **In this thesis .....**
