# Supplementary material for: Dynamics and control of typhoid fever in Sheno town, Ethiopia: A comprehensive nonlinear model for transmission analysis and effective intervention strategies
Source: PLoS One. 2024 Aug 7;19(8):e0306544. doi: 10.1371/journal.pone.0306544 (PMC11305579; doi:10.1371/journal.pone.0306544)
Supplement: Attachment — Submitted filename: Response 3.docx [file pone.0306544.s005.docx]

**Dear Reviewer #3,**

We extend our heartfelt appreciation for your thorough review of our manuscript and the invaluable feedback you provided. Your insights have been pivotal in refining our work to enhance its clarity, accessibility, and scientific merit. We have carefully considered all your suggestions and have made the following updates to enhance the clarity and scientific acceptance of our work:

1. **Simplification of Language:** We have carefully rephrased and simplified complex wording throughout the manuscript, particularly in ***the Abstract, Introduction, Model Formulation, numerical section and Recommendations sections***. This ensures that our findings are effectively communicated to a diverse audience.
2. **Expansion of References**: In response to your suggestion, we have enriched the references list by incorporating additional relevant research. Specifically, we have included the study titled "Quantitative Functional Evaluation of Liver Fibrosis in Mice with Dynamic Contrast-enhanced Photoacoustic Imaging" (Radiology, 300(1), 89-97. doi: 10.1148/radiol.2021204134). This addition strengthens the contextual framework of our study. ***Thank you for your nice suggestions regarding our manuscript. We have incorporated the concepts from the suggested references into both the introduction and model formulation sections. Your input has been instrumental in enriching our study.***
3. **Literature Update on Typhoid Fever**: We have updated the literature review on Typhoid Fever with the inclusion of the reference "Fractional order mathematical modeling of typhoid fever disease" (Results in Physics 32 (2022): 105044). This update provides a more comprehensive overview of the current research landscape in the field. ***Thank you for your insightful perspectives and recommendations regarding our model. We have integrated the suggested references and other relevant works into the recommendations section. The enhancements and modifications made to this work are invaluable.***

Finally, we would like to say that your thoughtful critiques and recommendations have significantly enriched our manuscript, making it more robust and aligned with the latest advancements in the field. We are truly grateful for your time, effort, and expertise in evaluating our work.

Warm regards,

Belela Samuel Kotola (PhD)
